# Dapagliflozin targets the crosstalk between apoptosis, autophagy, and Hedgehog signaling pathways through AMPK activation in the adjuvant-induced arthritic rat model

**DOI:** 10.1007/s10787-025-01750-w

**Published:** 2025-05-12

**Authors:** Aya A. El-Demerdash, Samar F. Darwish, Marwa O. El-Derany, Ebtehal El-Demerdash

**Affiliations:** 1 Pharmacology & Toxicology Department, Badr University in Cairo (BUC), Badr City, 11829 Cairo Egypt; 2 Biochemistry Department, Faculty of Pharmacy, Ain Shams University, Cairo, 11566 Egypt; 3 Pharmacology & Toxicology Department, Faculty of Pharmacy, Ain Shams University, Abasia,, Cairo 11566 Egypt

**Keywords:** Rheumatoid arthritis, Dapagliflozin, Autophagy, Apoptosis, AMPK, And Hedgehog-signaling pathway

## Abstract

Rheumatoid arthritis is a long-term autoimmune disorder, causes joint capsule, cartilage, and bone damage. Dapagliflozin, a novel antidiabetic drug, demonstrated promising effects against different disorders. Herein, we aimed to detect the dose-dependent antiarthritic impact of dapagliflozin alone and in combination with methotrexate standard treatment. Complete Freund’s adjuvant-induced arthritic rats were treated with three doses of dapagliflozin (1, 5, or 10 mg/kg/day, p.o.) for 3 weeks, in which 10 mg dose showed eminent anti-arthritic effects according to gait score, paw diameter, arthritic index (AI), morphological and histological results. To reveal dapagliflozin mechanism, locomotor, biochemical, and histological measures were assessed in dapagliflozin (10 mg/kg/day) and/or methotrexate (0.75 mg/kg/week, i.p.)-treated arthritic rats. Radiography and histology confirmed the prominent anti-arthritic effect of dapagliflozin via reduced RF, MMP-1, and MMP-3, and improved gait score, ankle diameter, and AI. Anti-inflammatory impact was confirmed by the downregulation of TNF-α, IL-1β, IL-6, and NF-κb p65 expression. Upregulation of autophagy was detected through; Beclin-1, ULK-1, and ATG-7, in dapagliflozin treated arthritic rats. Furtherly, dapagliflozin stimulated apoptotic activity, by boosting articular levels of CASP-3, CASP-9, cartilage gene expression of p53, and Bax/Bcl_2_ ratio. Interestingly, dapagliflozin upregulates p-AMPK/t-AMPK articular activity. Additionally, dapagliflozin inhibited the Hedgehog signaling pathway, through the downregulation of cartilage Shh, ptch1, Smo, and Gli-1 expression. Dapagliflozin/methotrexate combination therapy exhibited greater anti-arthritic benefits compared to methotrexate alone. These data highlight dapagliflozin as an anti-rheumatic drug, either alone or with methotrexate.

## Introduction

Rheumatoid arthritis (RA) is an autoimmune disorder associated with synovial inflammation, cartilage degeneration, bone erosion, and eventual joint degeneration. It is associated with a global incidence range from 0.24 to 1%, which varies according to region and country (Almoallim et al. 2021). Patients with RA suffer from physical disability and joint destruction, leading to early mortality and reduced quality of life. Although lots of studies have been conducted on RA, till now, the exact etiology and pathogenesis are still not clear. It has been linked to synovial hyperplasia, inflammatory cell infiltration, and apoptosis resistance (Wang et al. 2017).

Autophagy can be seen as a survival mechanism that helps eliminate unneeded and malfunctioning parts of the cell including damaged organelles and protein aggregates (Das et al. 2012). Recent studies have established a connection between autoimmune diseases like RA and malfunctioning autophagy. Suppressing autophagy in an experimental arthritic mouse model lowered the number of osteoclasts and other bone erosion symptoms, suggesting that autophagy is essential for the destruction of bone tissue (Vomero et al. 2018). Studies conducted on fibroblast-like synoviocytes (FLS) in RA showed resistance to cell death and upregulation of autophagic pathways, allowing them to multiply and invade, resulting in persistent inflammation and joint destruction (Karami et al. 2020a, b).

Aberrant modulation of signaling pathways in the joint has been associated with abnormal proliferation of FLS and excessive production of inflammatory cytokines (Liu et al. 2021). Various treatments can aid in alleviating symptoms and slowing the disease progression, including non-steroidal anti-inflammatory drugs, corticosteroids, and biological disease modifiers (Babaahmadi et al. 2023). Nevertheless, current anti-arthritic drugs have failed to provide a complete cure for RA (Prasad et al. 2023).

The Sonic Hedgehog (SHh) signaling pathway is an important part of the Hedgehog (Hh) protein family, that plays a crucial role in embryonic development, tissue maintenance, and stem cell upkeep (Liu et al. 2018). When the Hh ligand binds to the Patched 1 (Ptch-1) membrane receptor, it activates Smoothened (Smo), a G-protein coupled receptor-like protein. Downstream molecules of Smo then facilitate signaling transduction from the cytoplasm to the nucleus, activating the transcriptional factors Gli 1–3 (Li et al. 2023). Multiple human tumors have been linked to carcinogenesis and metastasis through the improper activation of Hedgehog signaling (Zhu et al. 2022a). Recent studies have shown that Hedgehog signaling is upregulated in RA patients' synovial tissue, encouraging the proliferation and migration of FLSs. However, the involvement of the downstream signal of Hedgehog pathway in RA, and the possibility of targeting this pathway as a treatment approach has not been fully studied (Li et al. 2015a, b; Wilson and Chuang 2010; Zhu et al. 2020a, b, c).

Dapagliflozin (DAPA) is an oral hypoglycaemic drug with sodium-glucose cotransporter-2 (SGLT2) inhibition activity. It works by preventing the kidneys from reabsorbing glucose, improving liver function, aiding in weight loss, lowering uric acid levels, and reducing blood pressure (Dhillon 2019; Shao et al. 2020; Association 2020). The anti-inflammatory action of DAPA has been documented in multiple investigations through different methods. According to Lee et al. (2020), treatment with DAPA 1 mg/kg/day for 8 weeks led to decreased macrophage infiltration, and lower expression levels of inflammatory microglial-1 markers, including TNF-a, IL-1ß, and IL-6 in a normoglycemic rabbit model of atherosclerosis. This suggests that DAPA has potential anti-inflammatory effects (ElMahdy et al. 2020).

Therefore, our study aimed to screen the therapeutic potential of different DAPA doses in rats with adjuvant-induced arthritis (AIA). The study also explored the underlying mechanisms of DAPA anti-arthritic events alone or combined with methotrexate (MTX), uncovering the possible crosstalk between the Hedgehog signaling pathway, apoptosis, and autophagy.

## Materials and methods

### Drugs and chemicals

DAPA, (AstraZeneca Pharmaceutical Co., USA) was suspended in 1% carboxymethylcellulose (CMC) immediately before use. MTX was purchased from (Mylan SAS Co., France). Complete Freund’s adjuvant (CFA) (Sigma-Aldrich Co., USA) contains 1 mg per milliliter of heat-killed Mycobacterium tuberculosis suspended in sterile mineral oil. All other chemicals were of the highest purity grade commercially available.

### Ethics statement

The experimental methods involving animals were carried out following the US National Institutes of Health's Guide for Care and Use of Laboratory Animals (NIH Publication No. 85–23, revised 2011) and were approved by Ain Shams University Faculty of Pharmacy Ethical Committee for the use of animal subjects, Cairo, Egypt, approval no. 146.

### Experimental animals

Male Spargue-Dawly rats, weighting (180–200 g), were obtained from Nile Co. for Pharmaceutical and Chemical Industries, Cairo, Egypt. Animals were kept accommodating under standard environmental conditions of the research facility (temperature of 25 °C, relative humidity 60%, 12 h. light/dark cycle) with free access to food and water for 1 week before assignment to the experimental protocol. There was a concerted attempt to reduce the number of animals used and alleviate their pain.

### Experimental design

#### Phase I: dose–response screening study

A preliminary dose–response study was performed to determine the optimal therapeutic dose of DAPA in which 30 rats were randomly allocated into 5 groups (*n* = 6) as follows:

(1) Control group**:** healthy non-arthritic rats were subcutaneously injected with 0.1 mL of paraffin oil into the plantar surface of left hind paws and orally received 0.5% CMC (Kabel et al. 2021). (2) AIA group**:** arthritic rats were orally administered with CMC. (3) AIA + DAPA 1mg group**:** arthritic rats treated with DAPA 1 mg/kg/day, p.o. (4) AIA + DAPA 5mg group**:** arthritic rats treated with DAPA 5 mg/kg/day p.o. (5) AIA + DAPA 10mg group**:** arthritic rats treated with DAPA 10 mg/kg/day p.o. The dose selection of DAPA was based on previous studies (Durak et al. 2018; Mohamed et al. 2019; Thongnak et al. 2020). Treatment regimens were initiated once the symptoms of arthritis appeared on the next day of induction and continued for three weeks. Dose–response effect of DAPA was determined according to gait scoring, paw diameter, AI, morphological, and histological examination. All assessments were conducted by blinded investigators.

#### Phase II: investigating DAPA therapeutic mechanism.

**Fig. 1:**
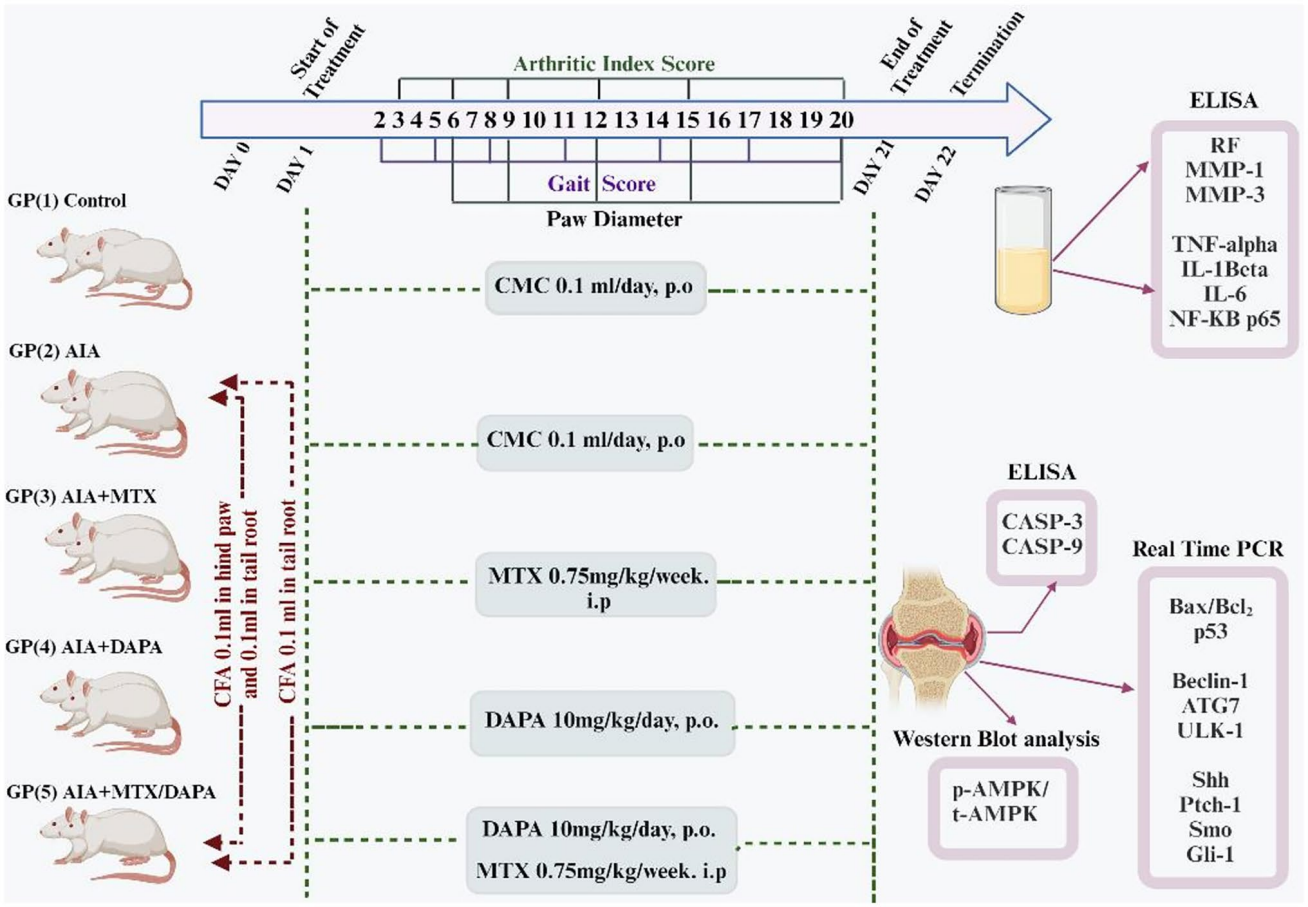
Experimental design summary showing the timeline for induction and drug administration of phase II

50 Rats were randomly allocated into 5 groups (*n* = 10) as follows:

(1) Control group: healthy non-arthritic rats were subcutaneously injected with 0.1 mL of paraffin oil into the plantar surface of rats’ left hind paws and orally treated with CMC. (2) AIA group, arthritic rats were orally treated with CMC. (3) MTX-treated group: arthritic rats were injected intraperitoneally with MTX (0.75 mg/kg/week for 3 weeks) (Paulos et al. 2006). (4) DAPA-treated group: arthritic rats were orally administered with the selected dose of DAPA from the phase I study (10 mg/kg/day). (5) MTX/DAPA-treated group: arthritic rats were cotreated with both MTX and DAPA. Arthritis symptoms were recorded the day after induction, and all therapy regimens were started and maintained for 3 weeks (Fig. 1).

At the end of the two experiments, blood samples were withdrawn from the retro-orbital plexus under anesthesia with thiopental (50 mg/kg) (El-Sahar et al. 2015) 24 h after the last dose, then centrifuged for 10 min and stored at − 20 °C for biomarker determination. After which, rats were sacrificed via decapitation. Cartilage and bone tissues were dissected out and either stored at − 80 °C for Real-Time Polymerase Chain Reaction (RT-PCR) analyses, homogenized at 1:10 (w:v) in potassium phosphate buffer (pH 7.5) for biochemical analyses, or fixed in 10% formalin for the preparation of paraffin blocks.

### Induction of adjuvant-induced arthritis (AIA)

The AIA model was induced by intradermal injection of 0.1 ml CFA into the plantar surface of rats’ left hind paws. On the same and the following day, a booster intradermal injection of 0.1 ml was administered into the root of the tail (Darwish et al. 2013).

### Evaluation of arthritis

#### Arthritic index

Arthritic pathological changes were recorded by a blind investigator using an AI score on days 3, 6, 9, 12, 15, and 20. Briefly, a scale of 0–4 was assigned, where 0 means no swelling; 1: swelling at the toe joint; 2: swelling at toe joint and sole; 3: swelling from toes to ankle joint; and 4: severe swelling of the entire paw and ankle joint. The AI score of each rat was determined via the sum of four limbs and the maximum score was 16. A successful induction of the model was determined by an arthritis index of more than 6 points (Wang et al. 2020b).

#### Gait score

Locomotor ability was determined every 3 days starting from day 2 by a blind investigator using a gait scoring system, which ranges from 0 to 3, where 0 = normal gait, 1 = slight lameness, 2 = lameness with weight-bearing on toes only, and 3 = non-weight-bearing lameness (Bush et al. 2001).

#### Hind paw diameter

Hind paw edema was evaluated using a digital caliper (Mitutoyo, Andover, UK) on days 6, 9, 12, 15, and 20 after CFA injection to monitor the degree of paw swelling till the end of treatment.

#### Radiographic examination

At the end of the experiments, anesthetized rats were placed on the radiological plate. Images of the hind paws were obtained using an X-ray instrument for veterinary use (model: ORANGE 1060HF, Brussel, Belgium) to evaluate the severity of arthritis. Radiographs were evaluated blindly for each rat's joint space narrowing, cartilage damage, and bone erosion. The changes were graded on a scale from 0 (normal), 1 (mild), 2 (moderate), and 3 (severe). The maximum potential radiological score for each rat was 6, which was determined by adding the scores from both hind paws (Cai et al. 2007).

### Histological evaluation

Paw joints were isolated after the animals’ sacrifice and fixed in 10% formalin for 24 h. Following that, the samples were decalcified in a 10% formic acid solution, embedded in paraffin, cut into 5 μm sections, and dyed with haematoxylin and eosin (H&E). Samples were examined using a light electric microscope (Olympus BX-50 Olympus Corporation, Tokyo, Japan) (Banchroft et al. 1996).

### Enzyme-linked immunosorbent assay (ELISA)

Following the manufacturer’s instructions, the following parameters were estimated in serum using rat ELISA kits: Rheumatoid factor (RF) (Cat No.: MBS880734**,** My BioSource, Inc., San Diego, USA), matrix metalloproteinase-1 (MMP-1) (Cat. No.: LS-F5522, lifespan BioScience, Inc., Seattle, USA), matrix metalloproteinase-3 (MMP-3) (Cat. No.: MBS729026, My BioSource, Inc., San Diego, USA), tumor necrosis factor-alpha (TNF-*α*) (Cat. No.: MBS355371, My BioSource, Inc., San Diego, USA), interleukin-1beta (IL-1β) (Cat. No.: MBS825017, My BioSource, Inc., San Diego, USA), interleukin -6 (IL-6) (Cat. No.: MBS355410, My BioSource, Inc., San Diego, USA), and nuclear factor kappa-B p65 (NF-κB p65) (Cat. No.: MBS733512, My BioSource, Inc., San Diego, USA).

Following the directions provided by the manufacturer, the following parameters were measured in articular tissues using rat ELISA kits: Caspase-3 (CASP-3) (Cat. No.: MBS261814, My BioSource, Inc., San Diego, USA), and Caspase-9 (CASP-9) (Cat. No.: E-EL-R0163, Elabsciences, USA).

### Western blotting analysis

Once the cartilage tissues had been homogenized in a cell lysis buffer, 20 μg of protein from each sample was loaded into an equal volume of 2 × Laemmli sample buffer. The samples were then separated using sodium dodecyl sulfate–polyacrylamide gel electrophoresis and transferred to a polyvinylidene difluoride membrane. Primary antibodies specific to AMP-activated protein kinase (AMPK) (Cat.No:2532, Cell Signaling Technology, Inc. USA) and p(Thr172)-AMPK (Cat.No: 2531, Cell Signaling Technology, Inc. USA) were used to block the membrane in a combination of tris-buffered saline with Tween 20 (TBST) buffer and 3% bovine serum albumin (BSA). After three 15-min washes with TBST, the membrane was incubated at 4 ^ο^C for 24 h. The membranes were incubated with horseradish peroxidase-conjugated secondary antibodies (Goat anti-rabbit IgG- HRP-1mg Goat mab -Novus Biologicals) for 1 h at room temperature. The quantitative protein band density was detected on the blot by enhanced chemiluminescent substrate (ClarityTM Western ECL substrate Bio-Rad Cat#170–5060). With the help of image analysis software, the band intensities of the phosphorylated and total target proteins were compared (normalization on the ChemiDoc MP imager) using β-actin as a reference control.

### Quantitative real-time PCR:

According to the manufacturer's protocol, total RNA was extracted from a joint homogenate of different groups using a High Pure RNA Isolation Kit (Cat. No. 11 828 665 001) (Roche Diagnostics GmbH, Germany). All primers were purchased from (Macrogen, Seoul, Republic of Korea). Primer sequences from Table 1 were used in real-time PCR.

**Table 1:** Primer sequences of RT-qPCR

Gene	Primer sequences
Bax	Forward: 5′-CAAGGCCCTGTGCACTAAAG-3′ Reverse: 5′-GTCACTGTCTGCCATGTGGG-3′
Bcl_2_	Forward: 5′-TCACAGAGGGGCTACGAGTG-3′ Reverse: 5′-CCGTAGAGGCGACGTCCTG-3′
P53	Forward: 5′-CCATCTACAAGAAGTCAC AACAC-3′ Reverse: 5′-CCCAGGACAGGCACAAAC3′
Beclin-1	Forward: 5′-CACCCACTGTGTGAGGAATG-3′ Reverse: 5′-TCCTCCAAGGCCAACTCCTT-3
ULK1	Forward: 5′- TCGAGTTCTCCCGCAAGG-3′ Reverse: 5′-CGTCTGAGACTTGGCGAGGT-3
ATG7	Forward: 5ʹ-CCAGTGACGCCAGATTTCC-3ʹ Reverse: 5ʹ-GGCAGGCACAGATGCTATG-3ʹ
Ch	Forward: TCCGATGTGTTCCGTTACC Reverse: AACCTTGCCTGCTGTTGC
Ptch1	Forward: CACCAAGTGATTGTGGAAGC Reverse: CTGTTGCCGAGAGTTCAAGG
Smo	Forward: ATGCGTGTTTCTTTGTGGGC Reverse: ACACAGGATAGGGTCTCGCT
Gli-1	Forward: AACTCCACGAGCACACAGG Reverse: GGCAGTCCGTCTCATACACA
β-actin	Forward: 5′-ACCACCATGTACCCAGGCATT-3′ Reverse: 5′-CCACACAGAGTACTTGCGCTCA-3′

Thermal cycling conditions for SYBR green RT-PCR were performed using Maxima SYBR Green/Fluorescein qPCR Master Mix (2X) Kit (Cat. No. K0241) (Thermo Fisher Scientific, Waltham, USA) as follows: primary denaturation for 8 min at 94 °C, followed by 40 cycles of amplification (secondary denaturation at 94 °C for 30 s, annealing at 56 °C for 45 s and extension at 72 °C for 50 s) with a final extension for 7 min at 72 °C. For MT2, samples were denatured for 8 min at 94 °C, followed by 40 cycles of 30 s denaturing at 94 °C, 45 s annealing at 58 °C, 30 s extensions at 72 °C, and a final extension of 7 min at 78°C.

The Stratagene MX3005P software Rotor-Gene Q was used to determine amplification curves and CT values. Using the following ratio: (2^−ΔΔct^), quantitative analysis was performed to determine the variation of gene expression on the RNA of the different samples according to the delta CT method (ΔΔCT) published by Livak and Schmittgen (2001).

### Statistical analysis

Parametric data were presented as mean ± SD with a p-value less than 0.05 representing significance. Comparisons of means were done using one-way analysis of variance (ANOVA), except for paw diameter analyzed by two-way ANOVA, followed by the Tukey–Kramer as a post-hoc test for multiple comparisons. Data were presented as the median and interquartile range, and non-parametric data was analyzed using the Kruskal–Wallis test and Dunn's multiple comparison tests. Statistical analysis and sketched graphs were performed using GraphPad Prism software version 8 (GraphPad Software, Inc., La Jolla, CA, USA).

## Results

### Phase I: the anti-arthritic effect of different DAPA doses

#### Effect on arthritic index

**Fig. 2:**
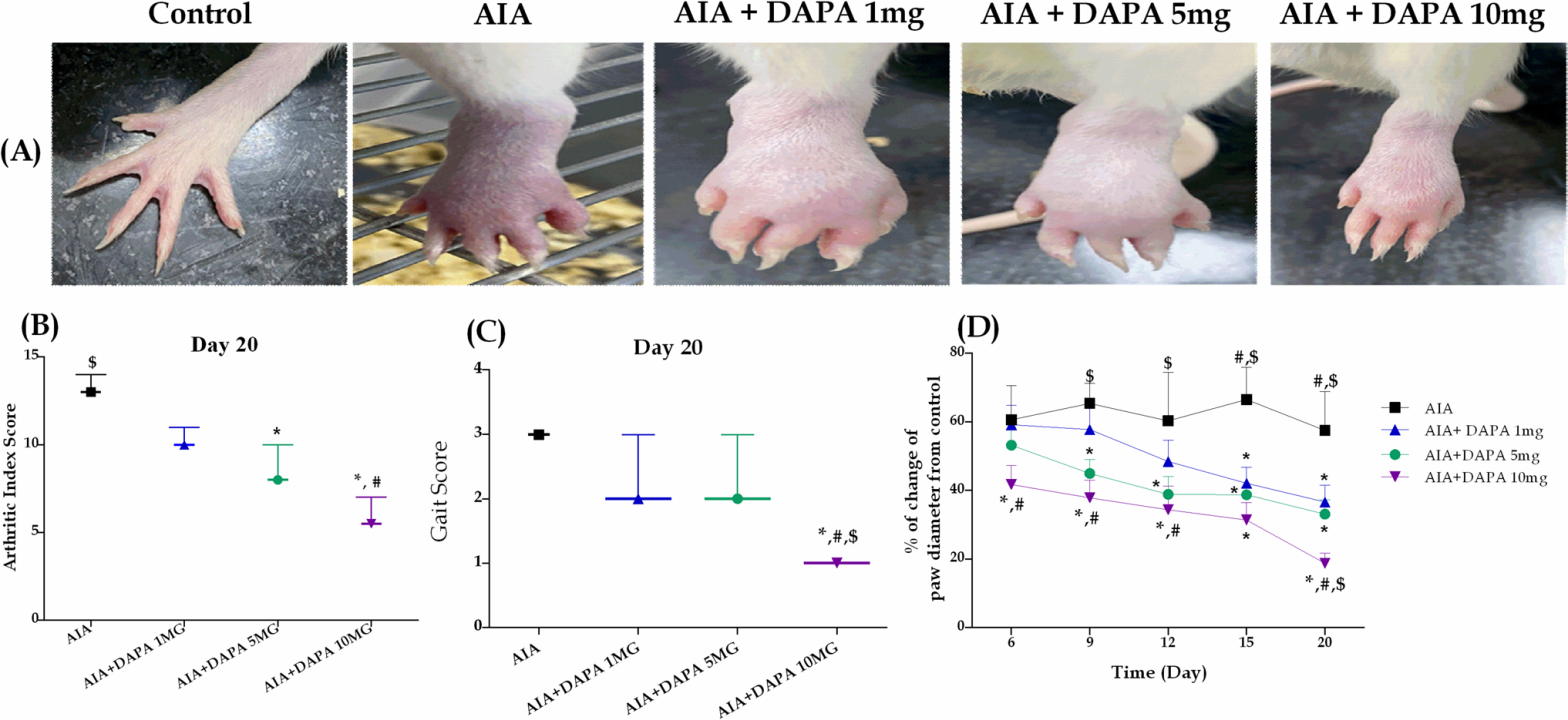
Effect of different doses of DAPA (1, 5, 10mg/kg/day) in CFA-induced arthritic rats. **A** Photographs of rats’ hind paws. **B** Arthritic index. **C** Gait Score. Data are represented as medians ± interquartile ranges (*n* = 10). *, # or $: significantly different from the corresponding AIA, DAPA 1mg or DAPA 5mg group, respectively at *p* ≤ 0.05 using the Kruskal–Wallis test followed by Dunn’s multiple comparison test. **D** Percentage change of paw diameter. Data are represented as mean ± SD. *, # or $: significantly different from the corresponding AIA, DAPA 1mg or DAPA 5mg group, respectively at *p* ≤ 0.05 using two-way ANOVA followed by Tukey–Kramer multiple comparison test

Concerning the arthritic index, the AIA rats, treated or non-treated, showed severe hind paws' edema starting from day 3 and continued to increase through the experiment compared to healthy non-arthritic rats (Fig. 2A). Administration of either of the three doses of DAPA improved the arthritic severity at the end of the experiment in a dose-dependent manner compared to non-treated rats. There was a marked improvement in the severity of arthritis in rats treated with either 5 or 10 mg/kg of DAPA compared to arthritic non-treated ones. Interestingly, treatment with DAPA 10 mg/kg/day demonstrated the most significant improvement in arthritic severity even when compared to the lowest dose (1 mg) (Fig. 2B).

#### Effect on gait score

Compared to healthy non-arthritic rats, the AIA group showed higher gait scores starting from day 2 and continued to increase throughout the experiment. Treatment with either DAPA 1 or 5 mg dose showed a slight non-significant reduction in gait score along the experiment compared to arthritic non-treated rats. On the contrary, screening revealed that only treatment with DAPA 10mg/kg showed significant improvement in gait score compared to arthritic non-treated rats. Furthermore, the highest dose of DAPA induced additional significant improvements in gait score in comparison with both the lowest doses of 1mg and 5mg-treated rats (Fig. 2C).

#### Effect on paw diameter

Based on caliper measurements of paw thickness, administration of CFA caused a substantial increase in paw diameter of all experimental groups except for the healthy non-arthritic rats. Treatment with different doses of DAPA showed a significant reduction in paw diameter in a dose-dependent manner compared to AIA-treated rats. Interestingly, treatment with DAPA 10 mg showed a superior reduction in paw swelling than 1mg DAPA starting from day 6 and continuing till the end of the experiment. Furthermore, the 10mg of DAPA exerted a significant reduction in paw diameter in comparison with the 5mg dose at the end of the experiment (Fig. 2D).

#### Histopathological examination

**Fig. 3:**
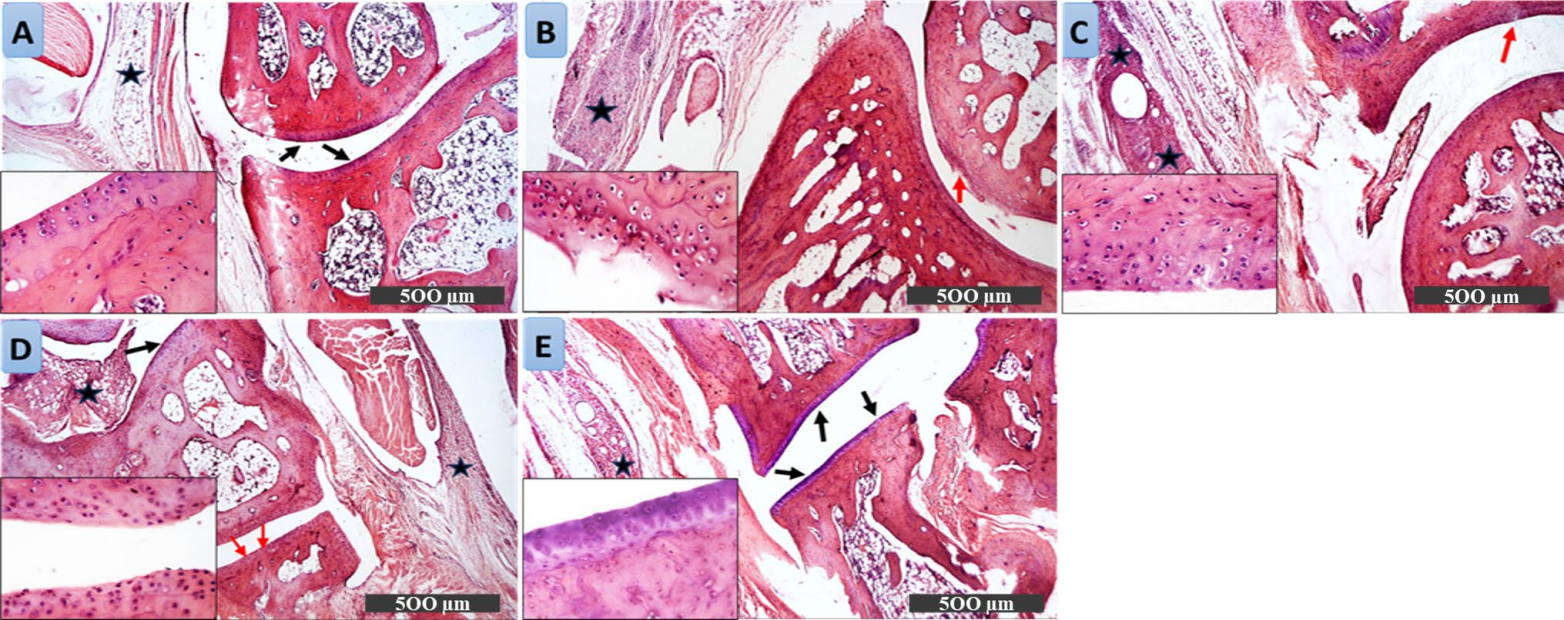
Photomicrographs of H&E-stained hind paw sections showing the effect of different doses of DAPA (1, 5, 10 mg/kg/day) in CFA-induced arthritic rats. **A** Control: showing normal articular cartilage structure, synovial membrane, well-organized chondrocytes, and intact homogenous intercellular matrix (arrow). **B** AIA: sections from arthritic non-treated rats showing articular surface erosions and fissures (red arrow) with degenerative changes of adjacent chondrocytes accompanied by severe mononuclear inflammatory cell aggregates in synovial membranes (star). **C** AIA + DAPA 1mg: section from arthritic rats treated with DAPA 1mg showing focal articular surface erosions, irregularities (red arrow), and loss of intercellular matrix staining intensity. Sever mononuclear inflammatory cells aggregate in synovial membranes (star). **D** AIA + DAPA 5 mg: section from arthritic rats treated with DAPA 5mg showing almost intact articular surfaces (black arrow) accompanied with moderate inflammatory cells infiltrating in synovial membranes and periarticular connective tissue (star). **E** AIA + DAPA 10mg: section from arthritic rat treated with DAPA 10mg with apparent intact articular surface without records of abnormal alterations (black arrow). Reduction of inflammatory cell infiltrates in synovial membranes and periarticular tissue (star) with normal articular cartilage structure

Rats' hind paw sections from the control group showed normal articular cartilage structure, well-organized chondrocytes, and intact homogenous intercellular matrix (Fig. 3A). On the contrary, sections from AIA rats showed articular surface erosions and fissures with degenerative changes of adjacent chondrocytes accompanied by severe mononuclear inflammatory cell aggregates in synovial membranes (Fig. 3B). The arthritic rats treated with DAPA 1 mg showed focal articular surface erosions, irregularities, loss of intercellular matrix staining intensity, and severe mononuclear inflammatory cells aggregate in synovial membranes (Fig. 3C). Treatment with DAPA 5 mg showed almost intact articular surfaces accompanied by moderate inflammatory cell infiltration in synovial membranes and periarticular connective tissue (Fig. 3D). Section from DAPA 10 mg-treated rats demonstrated apparent intact articular surface without records of abnormal alterations. Moreover, a significant reduction of inflammatory cell infiltrates in synovial membranes and periarticular tissue was observed showing normal articular cartilage structure (Fig. 3E).

### Phase II: mechanisms underlying the anti-arthritic effect of DAPA

According to the results of phase I, the dose of 10 mg of DAPA, which exerted superior anti-arthritic effects, was selected for further mechanistic investigation in comparison with the standard MTX treatment.

#### Effect on arthritic index

**Fig. 4:**
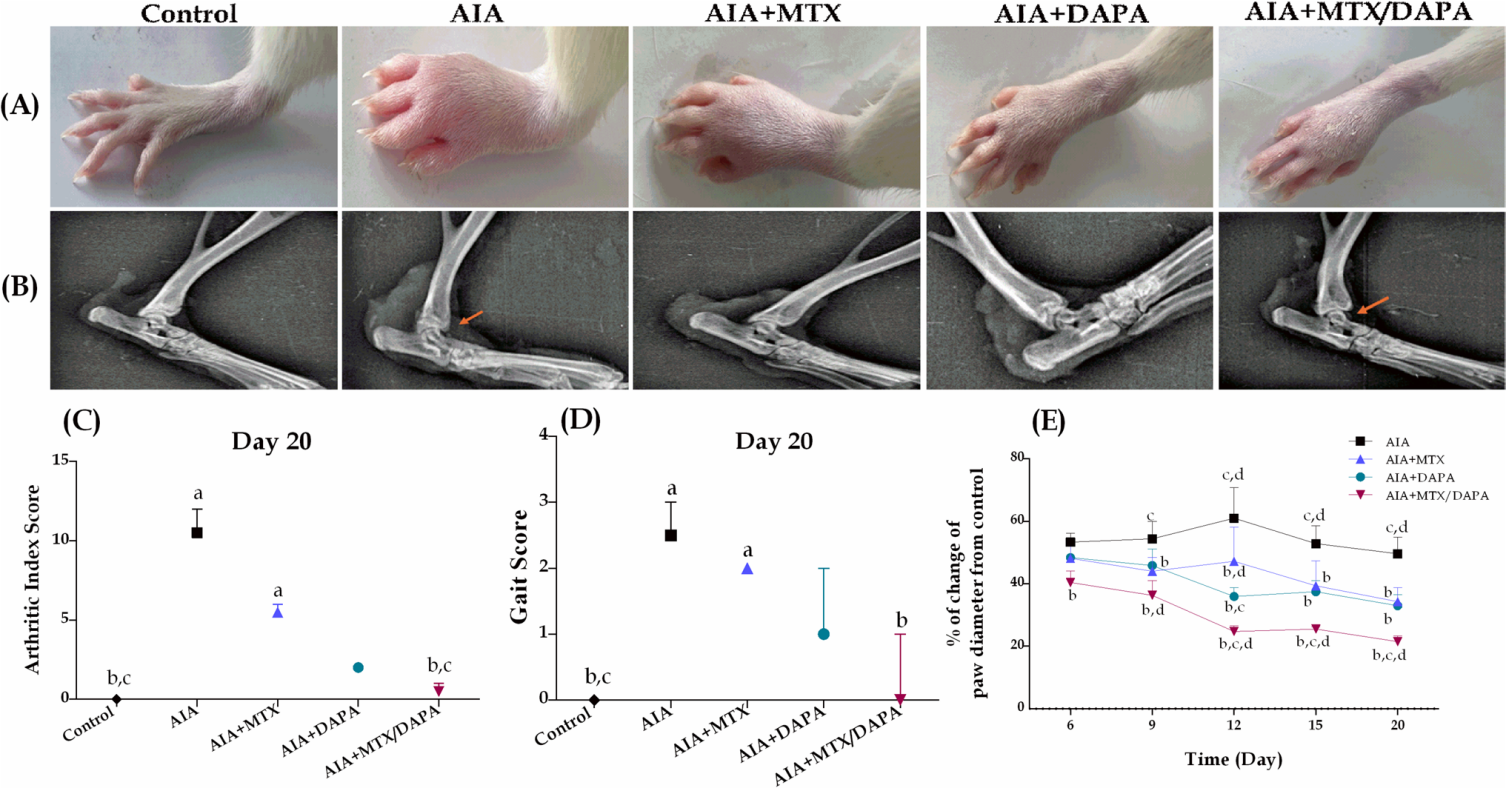
Effect of MTX and/or DAPA (10 mg/kg/day) in CFA-induced arthritic rats. **A** Photographs of the rat’s hind paws. **B** Radiographs of the rat’s hind paws. **C** Arthritic index. **D** Gait score. Data are represented as medians and interquartile ranges (*n* = 10). a, b, c, or d: Statistically significant from control, AIA, MTX, or DAPA-treated group, respectively at *p* ≤ 0.05 using the Kruskal–Wallis test followed by Dunn’s Multiple Comparison Test. **E** Percentage change in paw diameter. Data are represented as mean ± SD. a, b, c, or d: Statistically significant from control, AIA, MTX, or DAPA-treated group, respectively at *p* ≤ 0.05 using two-way ANOVA followed by Tukey–Kramer Multiple Comparison Test

The induction of arthritis with CFA injection resulted in severe hind paws' edema in treated or non-treated arthritic rats throughout the experiment as compared to healthy non-arthritic group (Fig. 4A). Treatment with either MTX or DAPA alone did not exhibit any significant difference from arthritic non-treated rats, although DAPA monotherapy succeeded in normalizing the arthritic index. Meanwhile, only the combination therapy of MTX with DAPA showed significant improvement in arthritic index, as compared to both arthritic non-treated rats and MTX-treated ones, in addition to restoring the arthritic severity to normal levels (Fig. 4C).

#### Effect on gait score

Administration of CFA showed a substantial increase in gait score in all CFA-treated groups starting from day 2 until the end of the study. Both AIA and MTX-treated groups boosted higher gait scores in comparison with the control group. Treatment with MTX alone exhibited no significant difference in gait score compared to arthritic non-treated rats. On the contrary, DAPA-treated rats showed considerable improvement by normalizing the gait score. Furthermore, in rats concurrently treated with DAPA and MTX, a significant decline in gait score was observed as compared to arthritic non-treated rats, and reached normal levels (Fig. 4D).

#### Effect on paw diameter

Based on caliper measurements of paw thickness, the hind paws of different treated groups revealed marked enlargements in comparison with healthy non-arthritic rats. Both sole treatments with either MTX or DAPA showed a significant reduction in paw diameter starting from day 9 till the end of the experiment, as compared to arthritic non-treated rats. In contrast, animals given a combination of MTX and DAPA showed a marked earlier reduction in paw diameter on day 6 and continuing until the end, as compared to AIA rats. Interestingly, only the combination therapy induced a superior reduction in paw diameter compared to both monotherapies of either DAPA or MTX, starting from day 12 till day 20 (Fig. 4E).

#### Radiological examination

X-ray imaging was performed on the hind paw to observe changes related to swelling, bone proliferation, and joint space reduction of different treatment groups. Remarkable changes were observed in arthritic non-treated rats after administration of CFA including soft tissue swelling, narrow joint space, and bone erosion. Treatment with either MTX or DAPA alone failed to effectively reduce the alterations mentioned above compared to the AIA group. On the contrary, concomitant treatment with DAPA and MTX substantially improved arthritic changes in joint structure, particularly bone proliferation, erosion, and joint space (Fig. 4B).

#### Histopathological examination

**Fig. 5:**
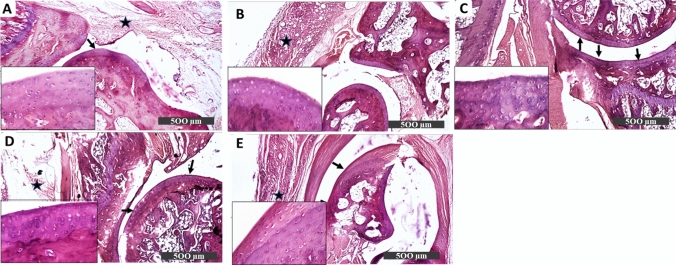
Photomicrographs of hind paw sections of arthritic rats treated with MTX and/or DAPA (10 mg/kg/day) stained by H&E. **A** Control: showing normal articular structure, synovial membrane, well-organized chondrocytes, and intact intercellular matrix. **B** AIA: showed multiple articular surface irregularities with marked diffuse inflammatory cell infiltrates in synovial membranes and periarticular connective tissue (star). **C** AIA + MTX: showed an apparent intact articular surface with intact chondrocytes and intercellular matrix (arrow). However, persistent records of severe inflammatory cell infiltrates in synovial membranes and periarticular connective tissue were observed (star). **D** AIA + DAPA: Showed intact articular surface with no abnormal alterations (black arrow), and a significant reduction in synovial membrane inflammatory cell infiltrates (star). **E** AIA + MTX/DAPA: showed an intact articular surface, intact chondrocytes all over deeper cartilaginous zones, and an intact intercellular matrix (arrow) with mild inflammatory cell infiltrates in synovial membranes (star)

The histopathological analysis of the control group's cartilage tissues revealed normal articular cartilage structures, with intact smooth surfaces. Well-organized intact chondrocytes inside lacunae, either single or grouped at different zones with intact homogenous intercellular matrix, were recorded. Intact synovial membranes and covering epithelium with minimal inflammatory cell infiltrates were also observed (Fig. 5A). Meanwhile, the AIA-treated group showed multiple focal articular surface erosions and irregularities accompanied by marked diffuse inflammatory cell infiltrates in synovial membranes and periarticular connective tissue with congested blood vessels (Fig. 5B).

The MTX-treated group showed an apparent intact articular surface with many figures of apparent intact chondrocytes all over most of the deeper cartilaginous zones with intact intercellular matrix. However, persistent records of severe inflammatory cell infiltrates were shown in synovial membranes and periarticular connective tissue with congested blood vessels (Fig. 5C). The DAPA-treated group showed apparent intact articular surfaces with many figures of apparent intact chondrocytes all over most of the deeper cartilaginous zones with intact intercellular matrix. Moreover, milder dissolution of inflammatory cell infiltrates in synovial membranes was noticed (Fig. 5D).

MTX/DAPA cotreated group demonstrated apparent intact articular surface without records of abnormal alterations. Moreover, a significant reduction of inflammatory cell infiltrates in synovial membranes and periarticular tissue was observed (Fig. 5E).

#### Effects on matrix metalloproteinases and rheumatoid factor

In CFA-treated rats, estimation of RF, MMP-1, and MMP-3 serum levels showed significant upregulation by 20, 3, and fivefold as compared to healthy non-arthritic rats. MTX treatment alone significantly reduced both matrix metalloproteinases and RF by 0.5-fold as compared to arthritic non-treated rats. In DAPA-treated rats, serum levels of RF, MMP-1, and MMP-3 were significantly reduced by 0.6, 0.7, and 0.7-fold, respectively, as compared to AIA rats. Additionally, treatment with DAPA alone showed marked decline in both matrix metalloproteinases and RF in comparison with MTX monotherapy. Concurrent treatment by MTX and DAPA induced further significant reduction in RF, MMP-1, and MMP-3 by 0.4-fold as compared to MTX alone. It is worth mentioning that arthritic rats treated with either MTX or DAPA alone displayed a significant difference in both matrix metalloproteinases and RF levels from healthy non-arthritic rats, while only the combination therapy normalized matrix metalloproteinases levels as shown in Table 2.

**Table 2:** Effect of MTX and/or DAPA (10 mg/kg/day) on serum levels of RF, and Matrix Metalloproteinases in CFA-induced arthritic rats

Group	RF (IU/ml)	MMP-1 (ng/ml)	MMP-3 (ng/ml)
Control	8 ± 0.85^b,c,d^	10.3 ± 0.72^b,c,d^	6.9 ± 0.93^b,c,d^
AIA	168 ± 19.5^a,c,d^	45.14 ± 4.9^a,c,d^	39.15 ± 4.3^a,c,d^
AIA + MTX	76.98 ± 12.6^a,b,d^	20.7 ± 1.9^a,b,d^	18.43 ± 2.6^a,b,d^
AIA + DAPA	60.08 ± 8.03^a,b,c^	15.4 ± 1.6^a,b,c^	12.12 ± 1.5^a,b,c^
AIA + MTX/DAPA	48.5 ± 6.02^a,b,c^	12.8 ± 0.95^b,c^	10.26 ± 1.3^b,c^

Data are presented as mean ± SD (*n* = 6). a, b, c or d: Statistically significant from control, AIA, MTX, or DAPA-treated group, respectively, at *p* ≤ 0.05 using one-way ANOVA followed by Tukey as a post hoc test. MTX; Methotrexate, DAPA; Dapagliflozin, RF; Rheumatoid factor

#### Effects on inflammatory markers

**Fig. 6:**
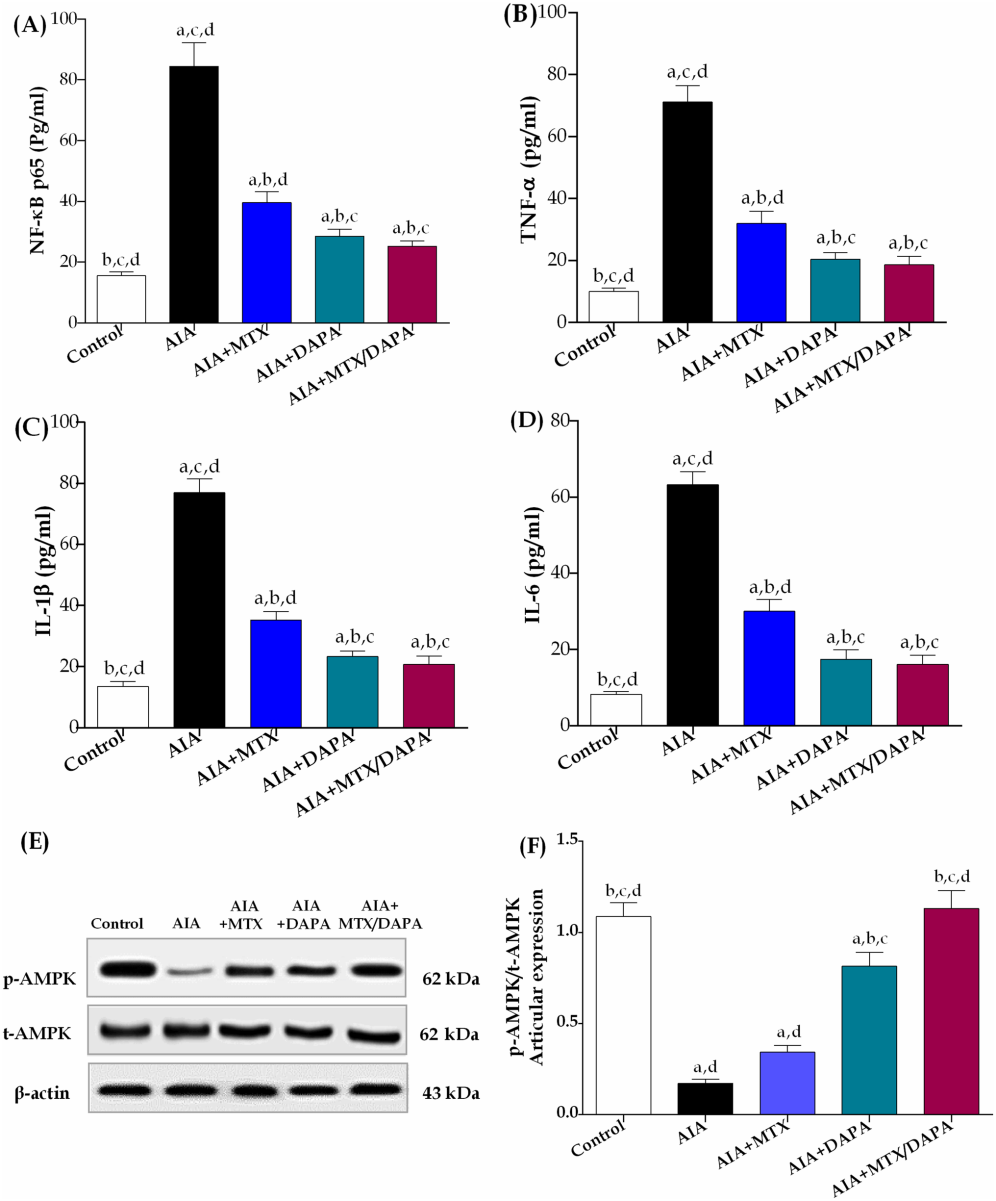
Effect of MTX and/or DAPA (10 mg/kg/day) on inflammatory markers in CFA-induced arthritic rats. **A** NF-κB p65 serum level, **B** TNF-α serum level, **C** IL-1β serum level, **D** IL-6 serum level, **E** Western blot bands of p-AMPK/t-AMPK articular protein expression. and **F** p-AMPK/t-AMPK graphical protein expression. Data are presented as mean ± SD (*n* = 6); a, b, c, or d: Statistically significant from control, AIA, MTX, or DAPA-treated group at *p* ≤ 0.05 using one-way ANOVA followed by Tukey–Kramer multiple comparison test. NF-κB; nuclear factor kappa B, TNF-α; tumor necrosis factor-alpha; IL-1β; interleukin-1β, and IL-6; interleukin-6; AMPK; AMP-activated protein kinase

All arthritic rats, including treated with either MTX and/or DAPA, displayed significant differences in all inflammatory markers from healthy non-arthritic rats. Assessment of serum NF-κB p65 and TNF-α levels revealed that arthritic non-treated rats showed a significant elevation by 444 and 606%, as compared to control group. Treatment with MTX alone significantly reduced serum levels of both NF-κB p65 and TNF-α by 53 and 55% as compared to arthritic non-treated rats. Interestingly, DAPA monotherapy showed significant downregulation in NF-κB p65 and TNF-α serum levels by 66 and 71% as compared to AIA-treated rats. Additionally, treatment with DAPA alone exerted a marked difference from treatment with MTX alone in both NF-κB p65 and TNF-α levels. When rats were given DAPA in combination with MTX, the levels of NF-κB p65 and TNF-α were 36 and 42% lower, respectively, compared to rats treated with MTX alone as shown in Fig. 6A and B.

Administration of CFA, significantly increased serum levels of IL-1β and IL-6 by 468 and 668%, respectively, compared to healthy non-arthritic rats. This significant increase in the inflammatory cytokines was markedly reduced in the MTX-treated rats by almost 54 and 53% in IL-1β and IL-6, respectively as compared to arthritic non-treated rats. Surprisingly, treatment with DAPA alone significantly attenuated serum levels of IL-1β and IL-6 by 70 and 72%, respectively, compared to AIA-treated rats, and induced further reduction in comparison with MTX monotherapy. However, MTX and DAPA cotreatment notably dropped the levels of IL-1β and IL-6 by 41 and 46%, respectively, compared to MTX sole treatment (Fig. 6C and D).

#### The effect on phosphorylated and total adenosine monophosphate-activated protein kinase (AMPK)

In Fig. 6E and F, AIA rats showed significant downregulation in p-AMPK/t-AMPK articular expression by 84% as compared to healthy non-arthritic rats. Treatment with MTX alone didn’t show any significant change in p-AMPK/t-AMPK expression from arthritic non-treated rats. On the contrary, compared to AIA-treated rats, DAPA monotherapy dramatically elevated p-AMPK/t-AMPK expression by 374%, and achieved further increment in comparison with MTX monotherapy. However, compared to MTX treatment alone, the articular expression of p-AMPK/t-AMPK was 230% higher in rats cotreated with DAPA. Noteworthy, only the combination therapy succeeded in normalizing p-AMPK/t-AMPK articular expression, and significant difference was noted from treatment with DAPA monotherapy.

#### Autophagy markers

All arthritic rats, including treated with either MTX and/or DAPA, displayed marked changes in all autophagy parameters compared to healthy non-arthritic rats. Rats intoxicated with CFA presented a statistically significant downregulation in Beclin1, ULK1, and ATG7 gene expression by 57, 35, and 41%, respectively, as compared to the control group. However, treatment with MTX alone significantly elevated the aforementioned autophagic markers by 51, 191, and 42%, respectively compared to arthritic non-treated rats.

**Fig. 7:**
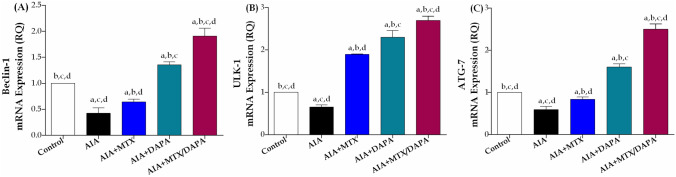
Effect of MTX and/or DAPA (10 mg/kg/day) on articular gene expression of autophagic markers in CFA-induced arthritic rats. Data are presented as mean ± SD (*n* = 6); a, b, c, or d: Statistically significant from control, AIA, MTX, or DAPA-treated group respectively, at *p* ≤ 0.05, using one-way ANOVA followed by Tukey–Kramer multiple comparison test

Interestingly, compared to AIA rats, the administration of DAPA, dramatically increased expression levels of Beclin1, ULK1, and ATG7 by 219, 253, and 171%, respectively. However, when DAPA was co-administrated with MTX, expression levels of Beclin1, ULK1, and ATG7 increased by 197, 42, and 199%, respectively, compared to rats treated with MTX alone. Furthermore, the combination therapy prompted additional escalations in all autophagic markers in comparison with using DAPA monotherapy (Fig. 7).

#### The effect on Hedgehog signaling pathway

**Fig. 8:**
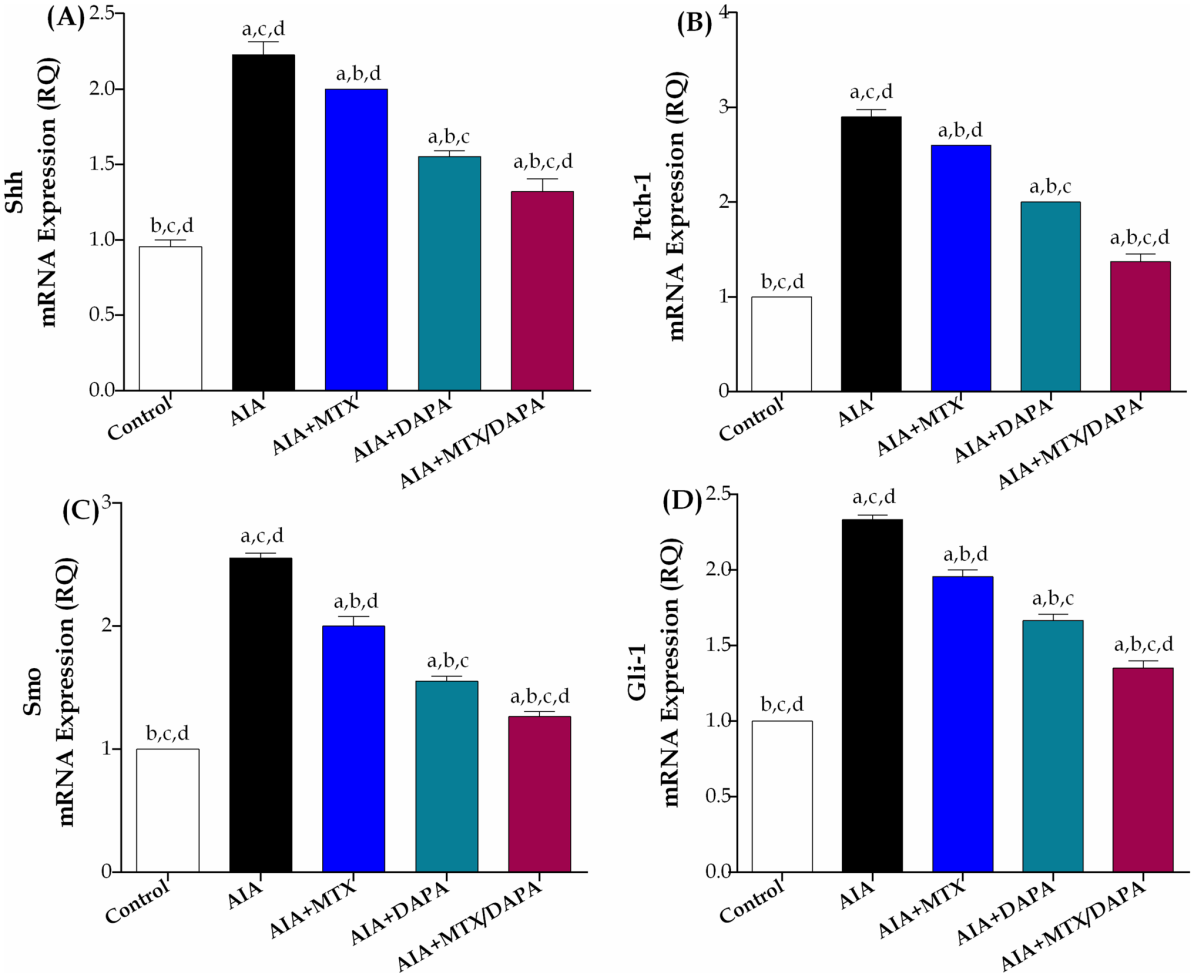
Effect of MTX and/or DAPA (10 mg/kg/day) on articular gene expression of Hedgehog signaling pathway markers in CFA-induced arthritic rats. Data are presented as mean ± SD (*n* = 6); a, b, c, or d: Statistically significant from control, AIA, MTX, or DAPA-treated group respectively, at *p* ≤ 0.05, using one-way ANOVA followed by Tukey–Kramer multiple comparison test

Induction of arthritis resulted in significant upregulation of Hedgehog signaling pathway genes in all groups correlated to the control one. SHh, Ptch1, Smo, and Gli-1 expressions in arthritic non-treated rats hiked by 133, 190, 155, and 133%, respectively, as compared to the healthy non-arthritic ones. Administration of MTX alone significantly reduced SHh, Ptch1, Smo, and Gli-1 articular gene expression by 10, 10, 22, and 16%, respectively, compared to the AIA-treated group. Compared to arthritic non-treated rats, monotherapy with DAPA considerably reduced the articular gene expression of SHh, Ptch1, Smo, and Gli-1 by 30, 31, 39, and 29%, respectively. It is worth noting that DAPA monotherapy showed further improvements in all autophagy genes expression over treatment with MYX alone. Interestingly, MTX and DAPA cotreatment significantly attenuated SHh, Ptch1, Smo, and Gli1 gene expression by 34, 47, 37, and 31%, respectively, compared to MTX alone, and additional increments were noted in all genes in relation to DAPA monotherapy as well (Fig. 8).

#### Apoptotic markers

**Fig. 9:**
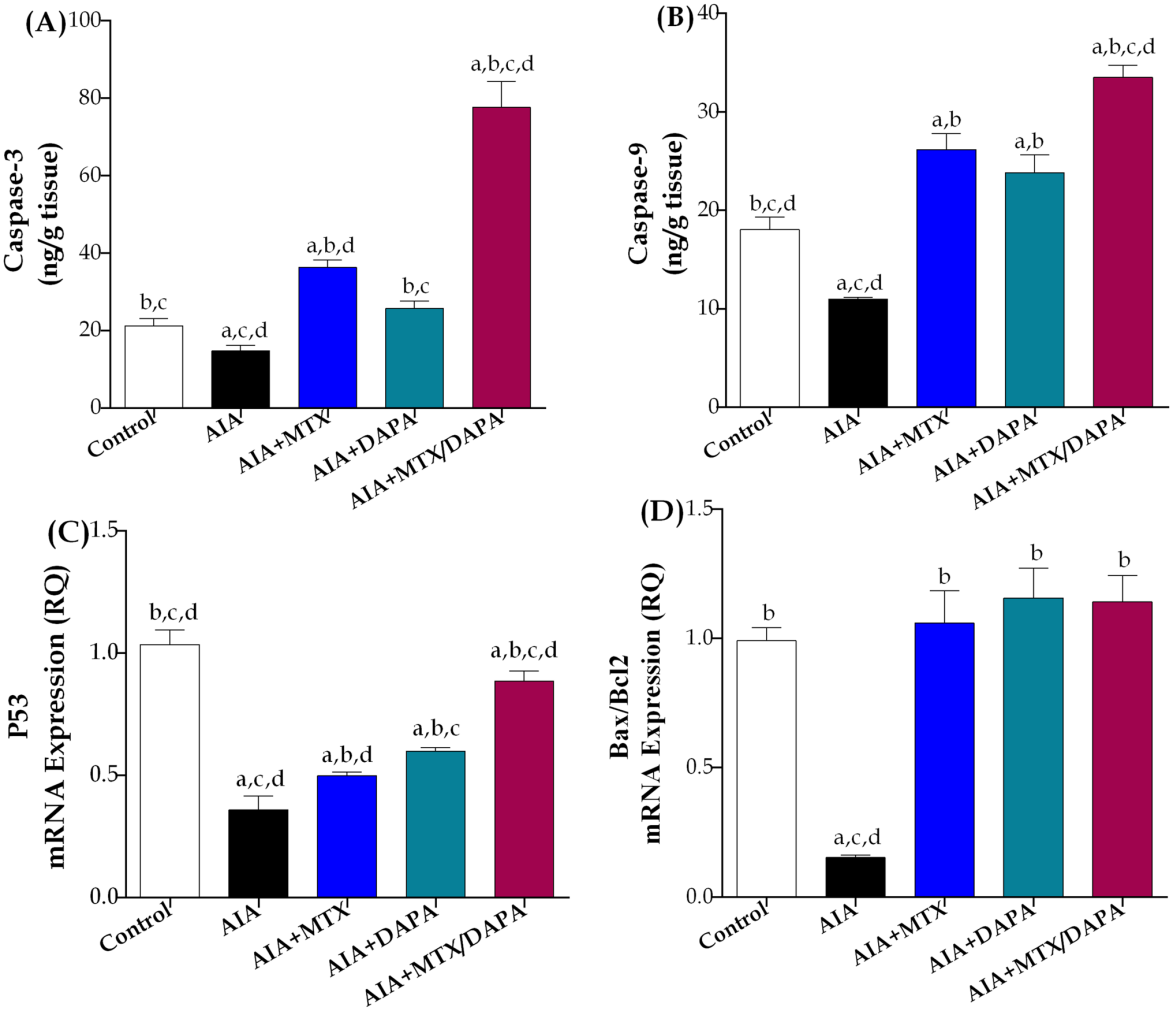
Effect of MTX and/or DAPA (10 mg/kg/day) on apoptotic markers and articular gene expression of p53 in CFA-induced arthritic rats. Data are presented as mean ± SD (*n* = 6); a, b, c, or d: Statistically significant from control, AIA, MTX, or DAPA-treated group, respectively, at *p* ≤ 0.05, using one-way ANOVA followed by Tukey–Kramer multiple comparison test

In comparison to the control group, the administration of CFA significantly changed the apoptotic markers expression in treated or non-treated rats. Attenuated articular levels of CASP-3 and 9 were noted in AIA group by 30 and 39%, respectively, compared to healthy non-arthritic rats. Treatment with MTX alone showed significant elevation in CASP-3 and CASP-9 by 146 and 138% respectively, as compared to arthritic non-treated rats. On the other hand, the group treated with only DAPA showed a significant increase in the levels of CASP-3 and CASP-9 by 74 and 117%, respectively, compared to the AIA-treated group. However, treatment with DAPA alone exerted significant changes in both caspases from MTX monotherapy and normalized CASP-3. When DAPA was co-administered along with MTX, the levels of CASP-3 and CASP-9 increased significantly by 114 and 28%, respectively, compared to MTX treatment alone, and further increments were observed in relation to DAPA alone (Fig. 9A and B).

Arthritic non-treated rats exhibited a 65% increase in P53 articular gene expression and an 85% decrease in Bax/Bcl_2_ ratio, compared to the control group. Upon treatment of arthritic rats with MTX, the expression of P53 gene increased by 39%, while the expression ratio of Bax/Bcl_2_ increased by 594%, compared to arthritic non-treated rats. Interestingly, compared to AIA-treated rats, administration of DAPA alone significantly increased p53 gene expression by 67% and Bax/Bcl_2_ ratio by 658%, and showed additional increase than MTX monotherapy in P53. Combining MTX with DAPA resulted in 78% and 48% marked rise in P53 gene expression compared to MTX or DAPA monotherapy. Noteworthy, all arthritic treated groups with MTX and/or DAPA succeeded in normalizing the expression ratio of Bax/Bcl_2_, while failed to normalize P53 (Fig. 9C and D).

## Discussion

RA is a lifelong condition associated with serious complications including progressive disability, and premature death. Its effects extend beyond the joints to include other body parts such as skin, eyes, lungs, and heart (Gad et al. 2021; Jang et al. 2022b). Current anti-rheumatic agents are only capable of managing symptoms and slowing down disease progression (Allen et al. 2018), which entails the continuous search for alternative treatments. DAPA has been reported in several studies to possess lots of pharmacological activities, including anti-inflammatory, apoptotic-modulation, and autophagic enhancement (Abdollahi et al. 2022; Anton et al. 2023; Arab et al. 2021; Deger et al. 2022; Jang et al. 2022a, b). In the present study, we introduced the possible therapeutic effects of DAPA against the AIA model in rats and the underlying mechanisms behind these outcomes, focusing on autophagy and Hedgehog pathways involvement.

In the current work, an animal model of arthritis was well established after 24 h of injecting CFA, in which treated and non-treated rats exhibited significant inflammatory and arthritic alternation, which were boosted by the successive injections of CFA. This was reflected by the weakened locomotion of AIA rats as a result of their hind paws' severe edema and the apparent degrees of redness, swelling, and limited activity. The arthritis induction was supported by histopathological analysis of articular tissues obtained from the arthritic non-treated rats which demonstrated multiple focal articular surface irregularities, accompanied by diffuse inflammatory cell infiltrates in synovial membranes and periarticular connective tissue. These findings were consistent with earlier studies showing similar deteriorated arthritic parameters in CFA-induced arthritis models (Gaafar et al. 2018; Gad et al. 2021).

As part of our research, we conducted a preliminary dose–response study to determine the potential therapeutic benefits of DAPA against AIA-model in rats using three different doses (1, 5, or 10 mg/kg/day). The lower doses of DAPA (1 or 5 mg) revealed slight alleviation in arthritic parameters, while the highest dose (10 mg) presented eminent antiarthritic effects of reduced paw diameter, gait score, and AI, compared to lower doses. This novel discovered effect of DAPA halted the multiple histopathological abnormalities incited by CFA in a dose-dependent manner, preserving almost normal intact articular surface upon using the highest dose, without apparent abnormal alterations nor inflammatory cell infiltrates. These results of our study provide the first evidence for the promising antiarthritic impact of DAPA in an adjuvant-induced rheumatoid arthritis rat model. Based on these notable findings, we were encouraged to conduct further investigations to unmask the underlying antiarthritic mechanism of DAPA, in favor of the maximal used dose (10 mg/kg). Consequently, the second research phase was carried out to ascertain the efficacy of DAPA in treating RA, either alone or in conjunction with MTX as an established RA standard medication, along with exploring the possible involvement of the Hedgehog pathway as a valuable area of interest.

CFA has been extensively used to induce arthritis in experimental animal studies which mimics human rheumatoid arthritis. In the present study, administration of CFA-induced arthritic alternation confirmed by elevated AI, observed paw inflammation and edema, increased gait score, and paw diameter compared to healthy non-arthritic rats. These results agreed with previous studies reporting the arthritic effects of CFA in rats and mice (Zhu et al. 2020a; Weng et al. 2021; Tran et al. 2023). According to our results, monotherapy of either MTX or DAPA slightly reduced both AI and gait score of arthritic rats, while notable improvements were found in paw diameter. The combination of DAPA with MTX supplemented a further enhancement in all arthritic indices, in comparison to both MTX or DAPA alone.

Upon radiographical inspection, administration of CFA displayed arthritic alternation in rats that appeared as soft tissue swelling, bone erosion, and joint space narrowing, in agreement with previous studies reporting CFA-arthritic radiographic effects (Patel et al. 2021; Sharma et al. 2023). Radiographic examination of the treated groups with MTX or DAPA showed no discernible inhibition of the joint abnormalities associated with arthritis. Meanwhile, concomitant treatment of DAPA with MTX significantly improved arthritic changes in joint structure, particularly erosion, and joint space which was confirmed by histological examination.

In RA, MMPs including MMP-1, MMP-3, MMP-8, and MMP-13 were found to be a crucial element for joint health, disease development, and progression (Araki and Mimura 2017; Bian et al. 2023). Prior clinical investigations reported that patients with RA have elevated serum and synovial fluid levels of MMP-1 and MMP-3 compared to osteoarthritis patients and normal ones, correlated to the levels of other disease activity markers (Abdelrahman et al. 2020; Hussein & Aboukhamis 2023; Li et al. 2022; Yoshihara et al. 2000). According to research, MMP-1 and MMP-3 played key roles in the pathogenesis of RA and have been suggested as measures of disease progression and joint destruction (Wang and Khalil 2018). More specifically, in early-stage RA non-treated patients, MMP-3 levels have been associated with the prediction of disease progression and treatment outcome (Yeo et al. 2022; Hussein and Aboukhamis 2023). It was found that elevated MMPs and positive RF have been associated with higher bone tissue destruction, chronic inflammation, more nodules, and multisystem involvement (Lin et al. 2020; van Delft and Huizinga 2020; Petrovská et al. 2021). In accordance, our study revealed that compared to healthy non-arthritic rats, CFA significantly elevated serum levels of MMP-1, MMP-3, and RF. Interestingly, treatment with 10 mg of DAPA alone showed a dramatic decrease in these elevated serum levels in arthritic rats. Furthermore, these critical rheumatoid severity markers were further reduced by the combined regimen of DAPA with the conventional MTX medication compared to both arthritic non-treated rats and MTX treatment alone.

Several immunological and inflammatory mechanisms are activated throughout the development of RA, which is described as an immune-mediated inflammatory process. Joint degeneration in RA is caused mainly by an imbalance between the pro- and anti-inflammatory cytokines release (Guo et al. 2018; Ding et al. 2023). The current data showed that CFA multiple injections induced a systemic inflammatory state in diseased rats, illustrated by the heightened serum levels of NF-κB, and the subsequent inflammatory cascade of IL-1β, IL-6, and TNF-α, in alignment with recent works (Nazir et al. 2023; Dar et al. 2023). According to previous reports, oral administration of DAPA (5 and 10 mg/kg) has shown an anti-inflammatory activity via suppression of matrix metalloproteinases levels with significant inhibition in IL-1β, IL-18, and TNF-α levels in lipopolysaccharide-mediated lung injury (Abd El-Fattah et al. 2022). On the same track, we revealed that treatment with DAPA (10 mg/kg/day) alleviated the convinced systemic inflammation in arthritic rats, shown by the downregulated levels of NF-κB and the successive cascade of TNF-α, IL-1β, and IL-6. Surprisingly, the accretion of DAPA to MTX standard therapy in the current study showed an additional reduction in serum levels of all inflammatory cytokines. This finding supports the prevalent anti-inflammatory impact of DAPA, which would benefit RA treatment.

Previous studies concerning possible remission of rheumatoid arthritis showed that activation of AMPK plays a significant role in regulating inflammation and disease progression. Accordingly, AMPK activation in RA models showed enhanced inflammatory response via reduction of IL-6, modulation of immune response, and attenuation of cartilage damage which could be linked to subsequence inhibition of Hedgehog pathway, responsible for FLS migration and pannus formation (Guma et al. 2015; Li et al. 2015a, b; Wang et al. 2022). AMPK activation has also been linked to the downregulation of several inflammatory mediators including TNF-α, IL-1β, IL-6, and NF-κB along with the restriction of Gli-1 transcription factor activation in different animal models (Wu et al. 2019; Wang et al. 2022). Indeed, multiple models have linked DAPA to increased AMPK activity. According to Faridvand et al. (2022), DAPA pretreatment significantly reduced high glucose-induced apoptosis and CASP-3 activity in human umbilical vein endothelial cells through increased p-AMPK. In the same context, our results showed that CFA administration constrained the articular phosphorylation activity of AMPK, which explains the heightened inflammatory state in arthritic non-treated rats. Meanwhile, treatment with DAPA showed dramatically increased AMPK phosphorylation activity in the articular tissue of arthritic rats. Surprisingly, DAPA had added such a benefit to MTX treatment, in which the latter failed to significantly activate AMPK phosphorylation. These confirmatory findings might disclose the impediment effect of DAPA on NF-κB and the successive cascade of TNF-α, IL-1β and IL-6. Consequently, DAPA certified itself as a substantial anti-inflammatory agent, which might repurpose DAPA as a promising treatment for systemic inflammatory disorders such as RA.

Studies on RA have shown that one of the key pathophysiological factors, especially in cases of synovial hyperplasia and pannus development, is the reduced apoptotic sensitivity of synovial fibroblasts. (Ding et al. 2023). It was reported that in RA joint, FLS showed cancer-like behavior, characterized by aberrant proliferation and resistance to apoptosis, which in turn induced persistent inflammation and tissue damage (Mousavi et al. 2021; Wu et al. 2021; Xi et al. 2022). In the current study, administration of CFA significantly downregulated articular levels of CASP-3 and CASP-9 as well as articular gene expression of p53 and Bax/Bcl_2_ in arthritic non-treated rats compared to healthy non-arthritic ones. Interestingly, in comparison to MTX-treated rats, treatment with DAPA alone significantly elevated cartilage tissue levels of CASP-3 and articular gene expression of p53 while there was no recorded difference in CASP-9 and Bax/Bcl_2_ ratio. Our results also showed that administration of DAPA in conjugation with MTX restored impaired apoptosis through a significant increase of CASP-3 and CASP-9 articular levels as well as elevated p53 articular gene expression compared to MTX treatment alone. In contrast to untreated arthritic rats, the Bax/Bcl_2_ expression was substantially increased in the combined DAPA/MTX-treated rats. These findings agreed with earlier research, reporting DAPA therapeutic impact on a mouse solid tumor model via improving the survival rate, and CASP-3 activities (Kabel et al. 2021).

In RA, autophagy has been found to play a dual role both protective and pathogenic depending on the cell type and disease stage (Vyawahare et al. 2022; Liu et al. 2023). Previous studies reported that autophagy was involved in RA-FLS survival and resistance to apoptosis as well as T-cell activation, osteoclast differentiation, and bone resorption. On the contrary, autophagy was also linked to chondrocytes and cartilage homeostasis maintenance (Karami et al. 2020b; Guo et al. 2021). In the same context, recent studies have shown that induction of autophagy was associated with the suppression of arthritis severity and desirable therapeutic outcomes in vivo and in vitro (Fernández-Rodríguez et al. 2021; Wang et al. 2024; Yang et al. 2022).

According to Ibrahim et al. (2022a, b), DAPA demonstrated a prospective therapeutic benefit through modulating autophagy in an Alzheimer's rat model as evidenced by increased hippocampal gene expression of Beclin1. In our study, the administration of CFA dramatically lowered the articular gene expression of ULK-1, Beclin-1, and ATG-7 as compared to control rats, leading to significant downregulation in the autophagy process, supporting the hypothesis of autophagy involvement in hindering arthritis severity. With the same concept, our investigation revealed that treatment with DAPA activated the autophagy axis, as evidenced by notable elevation of articular autophagic markers ULK-1, Beclin-1, and ATG-7 gene expression. Moreover, the adjuvant therapeutic regimen of DAPA with MTX dramatically elevated articular gene expression of ULK-1, Beclin-1, and ATG-7 compared to either arthritic non-treated or MTX-treated groups. Collectively, in the current study, DAPA exerted a dual beneficial effect through enhancing autophagy as well as modulating aberrant apoptosis in the RA model in a similar behavior to its effect against tumor models. This precious impact of DAPA in restoring the haemostatic balance between apoptosis and autophagy pathways in RA would help to prevent aberrant and uncontrolled growth of diseased cells, which is the primary factor in the seriousness of arthritis, and degeneration of joints.

Hedgehog signaling pathway has been linked to RA pathogenesis, specifically through abnormal proliferation and invasion of FLS in the synovium (Su et al. 2022; Zhu et al. 2022b). Studies reported that aberrant activation of Hedgehog pathway in RA patients contributed to RA-FLS multiplication and migration in a tumor-like behavior leading to synovial inflammation, joint destruction, and pannus formation through different mechanisms including activation of MAPK/Jun N-terminal kinase pathway, MAPK/extracellular signal-regulated kinase pathway, and upregulation of MMP1 and MMP3 which plays a crucial role in FLS aggressive behavior (Zhu et al. 2020c; Mousavi et al. 2021). According to research, overexpressed Hedgehog signaling components including Ptch1, Smo, and Gli1 were found in RA synovial tissues and FLS cultures (Su et al. 2022; Wang et al. 2014). Following the same lines, our results demonstrated that arthritic non-treated rats significantly elevated Shh, Smo, ptch1, and Gli-1 articular gene expression. Fortunately, treatment with DAPA either alone or in conjugation with MTX dramatically suppressed articular gene overexpression of Shh, Smo, ptch1, and Gli-1 when compared to MTX alone. These results clarify that DAPA mitigates RA pathogenesis by inhibiting Hedgehog pathway overactivation, an important factor in the disease's progression, contributing to joint inflammation and cartilage destruction.

Targeting the crosstalk of signaling pathways in multiple disorders, notably RA, is a promising approach for the evolution of novel therapeutic agents. AMPK, the substantial regulator of the inflammatory cascade, plays a core role in the crosstalk between autophagy and apoptosis in different pathological states (Villanueva-Paz et al. 2016). AMPK is conceivably linked with different autophagy stages. On the other hand, it has been suggested that AMPK regulates cell apoptosis under stressful circumstances. The phosphorylation of AMPK subsequently leads to the stimulation of proapoptotic mediators p53 and Bax, thus encouraging cells to go through apoptosis (Villanueva-Paz et al. 2016). Furthermore, AMPK is also linked to the regulation of Hedgehog signal in previous studies. One of the SGLT2 inhibitors induced AMPK phosphorylation, which hindered the Hedgehog pathway expression, thus restoring apoptosis and preventing abnormal cancer cell proliferation (Xie et al. 2020).

By highlighting our findings, DAPA has strongly targeted AMPK, the key controller of the inflammatory cascade. DAPA halted the CFA-induced inflammatory state of NF-κB, and the downstream cascade of TNF-α, IL-1β, and IL-6. According to recent studies, DAPA has been reported to suppress inflammation and enhance autophagy through activation of AMPK phosphorylation in different animal models (Hassan et al. 2024; Ibrahim et al. 2022b; Luo et al. 2023). Herein, DAPA activated AMPK-phosphorylation with subsequent enhancement of the autophagy process, via elevated articular expressions of ULK-1, ATG-7, and Beclin-1. Our study revealed that DAPA-induced AMPK effect has led to restoring apoptosis in arthritic tissue, through CASP-3, CASP-9, and p53 articular activation, as well as boosting Bax/Bcl2 ratio. Additionally, DAPA-mediated phosphorylation of AMPK might engage in its inhibitory action of Hedgehog signal, illustrated by Gli-1, Smo, and ptch1 articular downregulation. All of these beneficial impacts of DAPA were reflected in the improvement of AI, gait score, and paw swelling, along with the reduced levels of RF, MMP-1, and MMP-3 arthritis markers. Therefore, DAPA improved the histopathological deteriorations in articular tissues exerted by CFA administration.

During the current study, we aimed to clarify the possible antiarthritic effects of DAPA monotherapy, in addition to the potential beneficial impact of combining DAPA with MTX as an established drug for RA treatment. MTX has been considered the most conventional therapy for RA since the 1980s till this day and is often called the first-line medication for RA treatment, regardless of the serious limitations associated with long-term use, such as liver damage, infection, and developing lymphoma. Therefore, MTX was used in this study as a standard therapeutic reference for the treatment of RA. The mechanism underlying MTX effectiveness in RA was previously described by several theories. These include antagonistic effects of folate, adenosine signaling, oxidative stress induction, reduction of adhesion molecules, modification of cytokine profiles, and suppression of inflammation. The anti-inflammatory outcome of MTX only referred to the upregulated adenosine levels (Friedman and Cronstein 2019). However, the impact of MTX on serious signaling pathways involved in RA, such as Hedgehog and autophagy, and the exact underlying anti-inflammatory molecular mechanism, was not clearly defined.

Previous studies concerning MTX reported significantly lowered blood levels of MMP-3 with no evidence of MMP-1 levels in RA patients (Green et al. 2003; Yeo et al. 2022). Upon comparing the antiarthritic mechanism between MTX and DAPA monotherapies, we found that DAPA significantly improved serum measures of MMPs and RF, leading to normalizing these markers in combination therapy. Curiously, DAPA monotherapy prevailed in superior actions in impeding inflammatory cascade in comparison to MTX monotherapy towards all the inflammatory mediators assessed in the current work, specifically through increased AMPK phosphorylation activity in the articular tissue of arthritic rats, adding such a benefit to MTX treatment, in which the latter failed to achieve it. Such advantage made MTX benefit from a combination regimen with DAPA, giving the combination therapy the superior anti-inflammatory impact over both monotherapies.

While most MTX studies in RA revealed activated JNK pathway and subsequent induction of p53 gene expressions, there are no precisely detailed studies associated with its effect over other apoptotic markers (Spurlock et al. 2011, 2012; Wang et al. 2020a). During the current study, DAPA achieved more prominent autophagic and apoptotic effects than MTX in CASP-3 and P53 articular expressions and all autophagic assessed parameters. Our analysis revealed that combining DAPA with MTX has earned MTX further autophagic and apoptotic activity against invasive RA tissues, targeting the two crossed pathways responsible for cartilage and joint degradation. The current result is considered a new finding for MTX autophagic activity, which came in contrast with a previous study that showed reduced mRNA and protein levels of autophagy-related genes of ATG (3, 5, 12), ULK1 and Beclin1 in MTX-treated arthritic rats (Sun et al. 2023).

A previous study reported the possible impact of MTX on the Hedgehog pathway when only combined with curcumin for osteosarcoma treatment, not for RA (Giliberti et al. 2024). Herein, DAPA monotherapy achieved dominant enhancement in this serious pathway in correlation with MTX alone. Additionally, combining DAPA with MTX gained both monotherapies further improvement in Hedgehog signal, supporting the previous study finding and offering a valuable therapeutic benefit via modulating the pathway responsible for the abnormal multiplication of FLS in RA.

In conclusion, our research revealed the first evidence for the ameliorative effects of DAPA against CFA-induced arthritic model in rats alone or adjuvant to conventional RA therapy. DAPA has certified itself as a multitarget agent by aiming at AMPK, the hallmark of the interplay between apoptosis and autophagy, as well as Hedgehog pathways. These newly discovered antiarthritic impacts of DAPA could be attributed to its anti-inflammatory, autophagic, and apoptotic modulation properties, interlined with curbing the Hedgehog signaling parameters. Nevertheless, we recommend further investigations on the possible corroboration of DAPA in RA patients and other related inflammatory disorders, as monotherapy or as adjuvant to the conventional regimens.

## Data Availability

The datasets generated during the current study are available from the corresponding author upon reasonable request.

## Abbreviations

AI: Arthritic index
AIA: Arthritic-induced arthritis
AMPK: AMP-activated protein kinase
Bax: Bcl-2-associated X protein
Bcl-2: B-cell lymphoma 2
CFA: Complete Freund’s adjuvant CMC, carboxymethylcellulose
DAPA: Dapagliflozin
ELISA: Enzyme-linked immunosorbent assay
FLS: Fibroblast-like synoviocytes
Gli-1: Glioma-associated oncogene homolog-1
H&E: Hematoxylin and eosin stain
i.p.: Intraperitoneal
IL-1β: Interleukin-1beta
IL-6: Interleukin -6
MMP-1: Matrix metalloproteinases-1
MMP-3: Matrix metalloproteinases-3
MTX: Methotrexate
NF-κB p65: Nuclear factor kappa-B p65
PBS: Phosphate buffer solution
Ptch-1: Patched 1
RA: Rheumatoid arthritis
RF: Rheumatoid factor
SHh: Sonic Hedgehog
Smo: Smoothened
TNF-*α*: Tumor necrosis factor-alpha
